# Impact of Three Natural Oily Extracts as Pulp Additives on the Mechanical, Optical, and Antifungal Properties of Paper Sheets Made from *Eucalyptus camaldulensis* and *Meryta sinclairii* Wood Branches

**DOI:** 10.3390/ma13061292

**Published:** 2020-03-12

**Authors:** Mohamed Z. M. Salem, Wael A. A. Abo Elgat, Ayman S. Taha, Yahia G. D. Fares, Hayssam M. Ali

**Affiliations:** 1 Forestry and Wood Technology Department, Faculty of Agriculture (EL-Shatby), Alexandria University, Alexandria 21545, Egypt; 2 Restoration Department, High Institute of Tourism, Hotel Management and Restoration, Abukir, Alexandria 21526, Egypt; watsat20@yahoo.com; 3 Conservation Department, Faculty of Archaeology, Aswan University, Aswan 81528, Egypt; aymansalahtaha82@yahoo.com; 4 Laboratory and Research, Misr Edfu Pulp Writing and Printing Paper Co. (MEPPCO), Aswan 81656, Egypt; yahyagml@yahoo.com; 5 Botany and Microbiology Department, College of Science, King Saud University, P.O. Box 2455, Riyadh 11451, Saudi Arabia

**Keywords:** antifungal activity, *Eucalyptus camaldulensis*, oil additives, *Meryta sinclairii*, wood branch pulp

## Abstract

In the pulp and paper industry, several studies have been done to improve and enhance the properties of the mechanical, optical, and antimicrobial activities of pulp produced with different additives. In the present study, pulp of wood branches (WBs) from *Eucalyptus camaldulensis* Dehnh. and *Meryta sinclairii* (Hook.f.) Seem. was treated with *n*-hexane oily extracts (HeOE) from *Melia azedarach* L. fruits, *Magnolia grandiflora* L. leaves, and *Sinapis alba* L. seeds as additives at concentrations of 1%, 3%, and 5% based on oven-dry pulp weight. Measured mechanical properties were higher in paper sheets made from *E. camaldulensis* than *M. sinclairii* WB pulp. The highest tensile index values were observed with *E. camaldulensis* WB pulp treated with 5% HeOEs of *S. alba* (33.90 N·m/g) and *M. grandiflora* (33.76 N·m/g) compared to control (32.10 N·m/g); the highest tear index with 5% HeOE of *S. alba* (4.11 mN·m^2^/g) compared to control (3.32 mN·m^2^/g); and the highest burst index with 5% HeOE of *S. alba* (4.11 kPa·m^2^/g) compared to control (3.08 kPa·m^2^/g). The highest double-fold number value (9) was observed with *E. camaldulensis* WB pulp treated with 5% HeOEs of *S. alba*, *M. azedarach*, and *M. grandiflora* but with no significant difference compared to control treatment (8.33) or other HeOE treatments with *E. camaldulensis* WB pulp. Scanning electron microscope (SEM) examination showed clear inhibition of the growth of *Aspergillus terreus* with WB pulp paper discs of *E. camaldulensis* and *M. sinclairii* treated with HeOEs of *M. azedarach*, *S. alba*, and *M. grandiflora* at 3% and 5% compared to control treatment, while HeOEs at 5% concentration showed no growth of *A. niger* and *A. terreus*. The present findings establish that the HeOEs from *M. azedarach*, *S. alba*, and *M. grandiflora* at 3% and 5% are novel natural products that can be used as alternatives to improve the properties and antifungal activity of WB pulp produced from *E. camaldulensis* and *M. sinclairii*.

## 1. Introduction

With increasing worldwide demand for paper products that are of high quality and environmentally friendly, several studies searching for new materials and different pulp additives have been conducted. Pulp additives have been discussed broadly in several recently published works to show their effects on improving mechanical and physical properties as well as producing antimicrobial paper sheets [1,2,3,4,5,6,7,8].

Pulp additives have potential effects on the properties of pulp paper; for example, mechanical and fungal inhibition properties of paper sheets produced from linen fiber pulp with *Pinus rigida* Mill. ground wood (80 mesh) were enhanced [6], and positive effects against the growth of *A. terreus* and *A. niger* were observed. The addition of chitosan, cationic starch, or poly vinyl alcohol to pulp of old corrugated cartons or bagasse fibers enhanced the strength properties of the produced paper sheets [1,2]. The addition of cellulose nanofibrils in combination with cationic starch to bagasse pulp enhanced handsheet in terms of structural, optical, and strength properties [4]. Wheat straw and bagasse added prior to banana stem pulping enhanced the physical strength properties of paper sheets [3]. Mechanical, optical, and antifungal properties of pretreated papyrus strips with some nanomaterials and natural extracts were enhanced [5]. Chips of mixed hardwood followed by kraft pulping and pretreatment with black liquor improved tensile, tear, and burst indices [8].

Pulp of Whatman paper, cotton paper, and chemical pulp without additives showed decaying symptoms as infected with *Paecilomyces variotii* and *Trichoderma harzianum* [9]. Mechanical and optical properties of papyrus strips treated with some nanoparticles were enhanced, as well as inhibition against fungal infestation of *A. flavus*, *A. niger*, and *C. gloeosporioides* [5]. With the addition of particles (80-mesh) of *P. rigida* wood, *Costus speciosus* (J.Koenig) Sm. rhizomes, and *Senegalia catechu* (L.f.) P.J.H.Hurter & Mabb. rhizomes to linen pulp, the mechanical properties of handsheets were improved [6]. Flax paper sheets produced from pulp treated with chitosan + ZnO nanoparticle (NP) (1%) inhibited the growth of *A. flavus*, and chitosan + Ag NP (1%) inhibited *A. terreus*, while pulp treated with Paraloid B-72 + Ag NP (1%) showed the highest activity against *Stemphylium solani* [7].

Natural oils extracted from medicinal and aromatic plants have wide uses in different applications, for their antibacterial, antifungal, antioxidant, and insecticidal properties [10,11,12,13,14,15,16,17,18]. Fixed oils can be used as sources of unsaturated fatty acids [19,20] and for wood protection [18,21].

Oils from mustard (*Sinapis alba* L.) contain many glucosinolates [22], which possess potent biocidal activity against certain microorganisms [23]. Mustard oil has a special fatty acid composition; it contains oleic, linoleic, linolenic, and erucic acids [24,25,26]. At concentrations of 135 and 270 μL/L, essential oils from some herbs and spices, including mustard, added to the atmosphere packaging of rye bread delayed/inhibited the growth of *Penicillium roqueforti*, *P. corylophilum*, *A. flavus*, and *Eurotium repens* [27].

Oils from *Melia azedarach* L. showed potential bioactivity as insecticide [28,29]. Methanolic fruit extract of *M. azedarach* fruits and its various fractions showed antifungal activity against *Sclerotium rolfsii* [30]. Ethanol extract of ripe *M. azedarach* fruits showed potential antifungal activity against *A. flavus*, *Fusarium moniliforme*, *Microsporum canis*, and *Candida albicans* [31]. Extracts from *M. azedarach* flowers showed good antifungal activity against three fungi, *A. niger*, *A. fumigatus*, and *A. flavus* [32]. Essential oils of leaves, flowers, and seeds showed fungal growth inhibition against *F. oxysporum* and *C. albicans* strains [33]. In addition, ethanolic leaf extract of *M. azedarach* showed potential antifungal activity [34].

Most of the studies on leaf oil of *Magnolia grandiflora* L. were carried out on the essential oil, in which (*Z*)-*β*-ocimene (15.2%), *β*-bisabolene (13.3%), and germacrene A (12.9%) were the main compounds [35]. *M. grandiflora* oil with bornyl acetate (20.9%) as the main compound displayed antifungal activity against five dermatophyte strains [36].

During paper manufacture, fungal decay causes a negative economic impact on the produced pulp [37]. However, in the humid conditions, paperboard as well as paper used for packaging materials can be affected by the mold infestation and led to decrease the impact of the packaged product [38,39,40,41]. These led to use some treatments to increase the antimicrobial activity of paper pulp, i.e., for using of paper applications in the hygiene and medical sectors, the presence of chitosan in paper pulp inhibited the growth of *Staphylococcus aureus* and both fungi *Candida albicans* and *C. glabrata* [42,43] or for coatings in the textile industry [44]. Also, other study showed that the addition of Ag/ZnO composite paper and ZnO particles to paper as a filler was observed good antifungal activity against *A. niger* and *T. viride* [45].This study was aimed at evaluating the effects of natural oils extracted from *S. alba* seeds, *M. azedarach* fruits, and *M. grandiflora* leaves on the mechanical properties and fungal inhibition of paper sheets produced from the WB pulp of *E. camaldulensis* and *M. sinclairii*.

## 2. Materials and Methods 

### 2.1. Preparation of Wood-Branch Shavings 

Branches of *E. camaldulensis* and *M. sinclairii* were collected from Alexandria, Egypt, during January 2018. Green branches (moisture content (MC) ranging from 70% to 80%) with diameters ranging from 14 to 16 cm were cut into small logs (3 cm in thickness) and air-dried at room temperature for about 3 months at relative humidity (RH) of 65 ± 5%, then debarked, and the wood branches (WBs) were turned into shavings at a workshop mill at Alexandria City, Egypt. The sizes of shavings were measured, ranging between 2 and 2.5 cm, then screened to remove fine or dust particles. For characterization of raw materials, air-dried WB samples from *E. camaldulensis* and *M. sinclairii* were prepared in dimensions of 2 cm × 2 cm × l.5 cm [46,47], then the specific gravity (SG_0_) of each WB sample was determined based on the oven-dry (OD) weight and volume using the dimensions method [48].

Wood moisture content (MC) was measured based on the OD weight. A piece of wood was initially weighed and then dried in an oven at 103 ± 2 °C. The drying continued until the wood piece was completely dried, and this oven-dry weight was recorded. MC was calculated as follows: M = [(w − d)/d] × 100, where M is initial moisture content, W is initial wet weight (g), and d is OD weight (g). Five samples of WB were used for each measurement.

The percentages of moisture content (MC %) of wood branches (WBs) were 11.66% and 10.81%, while the specific gravity (SG_0_) values were 0.67 and 0.52 for WB of *E. camaldulensis* and *M. sinclairii,* respectively.

### 2.2. Chemical Analysis of Wood Branch Shavings 

For chemical analysis, the air-dried WB shavings were milled into a powder in a laboratory Wiley mill (A-47054, Weverk, Karlstad, Sweden), and a fraction passing through −40 mesh size but retained on +60 mesh size was used for chemical analysis. Chemical characterizations were carried out as per TAPPI Standard Test methods. 

#### 2.2.1. Solubility in Cold and Hot Water

For cold water solubility (T207 cm-08), 2 g of air-dried material meal was placed in a beaker (400 mL) and 300 mL of distilled water (DW) was slowly added. The extraction was done at 23 ± 2 °C with constant stirring for 48 h. The materials were transferred to a tare filtering crucible previously dried at 105 ± 2 °C to a constant weight, then washed with 200 mL of cold DW and dried to constant weight at 105 ± 2 °C. The heated crucible with a loosely stoppered weighting bottle was placed in a desiccator to cool before being weighed. For hot water extraction, 2 g of the specimen was transferred to a flask (250 mL) and 100 mL of hot distilled water was added, and it was placed in a boiling water bath then attached to a reflux condenser and digested for 3 h. After that, the contents of the flask were transferred to a previously dried tare filtering crucible then washed with 200 mL of hot water and dried to a constant weight at 105 ± 2 °C. The percentages of hot and cold water solubility were calculated using the following formula: hot or cold water solubility % = [(A − B)/A] × 100, where A is the OD weight of the test specimen before extraction (g), and B is the OD weight of the test specimen after extraction (g).

#### 2.2.2. Alcohol-Benzene Extractive Determination (T204 cm-17)

The amount of solvent-soluble nonvolatile material in the raw material, ethanol-benzene in a 1:2 mixture was used in a Soxhlet flask. A test specimen of 4 g (−40/+60 mesh) was put into position in the thimbles of the Soxhlet apparatus, then the extraction flask was filled with 150 mL of solvent mixture. The heating process was adjusted to provide a boiling rate for 6–8 h. After that, the solvent was evaporated with a rotary evaporator and the flask with its contents was dried in an oven at 105 ± 2 °C for 1 h, then cooled and weighed. The extracted content was determined as follows: extractive content % = [(W_1_ − W_2_)/W_3_] × 100, where W_1_ is the OD weight of the extract (g), W_2_ is the oven-dry weight of the blank residue (g), and W_3_ is the OD weight of the raw material (g).

#### 2.2.3. Acid-Insoluble Lignin (T222 om-15)

One g of OD powered sample extracted previously with ethanol-benzene was put in a beaker (500 mL) was extracted with solvent, then 300 mL hot water was added. Then 5 mL of 72% H_2_SO_4_ was added gradually at a temperature of 10–15 °C in small increments while stirring the sample with a glass rod. The beaker was kept in a bath at 2 ± 1 °C during dispersion of the material. After that, the beaker was covered with a watch glass and kept in a bath at 20 °C for 2 h, stirring the sample from time to time. The contents of the beaker were transferred to a flask and diluted to 3% H_2_SO_4_ with the addition of 575 mL of DW. The solution was boiled for 4 h under refluxing and the insoluble lignin was allowed to settle, then it was filtered, washed with hot water, dried at 105 °C in an oven to constant weight, cooled in a desiccator, and weighed. The lignin content was calculated as follows: lignin % = (A/W) × 100, where A is the weight of lignin (g) and W is the OD of the test specimen (g).

#### 2.2.4. Pentosans Determination (T223 cm-10)

In a boiling flask, 1 g of OD powdered sample was placed and 20 g sodium chloride, 100 mL of (3.85 N) hydrochloric acid, and few boiling stones were added. The flask was connected to the distillation apparatus and the acid level was marked, with the addition of 250 mL of (3.85 N) hydrochloric acid to the separator funnel. Heat was applied and the acid was distilled at 2.5 mL/min. The distillate was collected in a 250 mL volumetric flask with immersion in an ice bath. The distillation was conditioned for 90 min, and 360 ± 10 mL of distillate was collected, then 40 mL of filtered phloroglucinol solution that had previously been prepared for 1 week was added, and the mixture was allowed to stand for 16 h. Furfural phloroglucide (FP) was collected in a weight filtration crucible that had a thick asbestos mat. The precipitate was washed with 150 mL of cold water then dried for 2.30 h at 105 °C, cooled and weighed, and the increase in weight was considered to be FP. The weight of pentosans corresponding to the weight of FP was calculated as follows: pentosans = (a + 0.0052) × f × 100/m, where a is the weight of FP (0.157 and 0.18 g from *E. camaldulensis* and *M. sinclairii* WB, respectively), f = 0.887, a is the weight of the insoluble portion (mg), and m is the weight of the OD WB shavings (g).

#### 2.2.5. Holocellulose Determination by Chlorination Method

A 2.5 g extractive-free dried wood sample was placed in a 250 mL Erlenmeyer flask. Then 80 mL of hot DW, 0.5 mL acetone, and 1 g of sodium chlorite (NaClO_2_) were added. The mixture was heated in a water bath at 70 °C for 1 h. After this, another dose of 0.5 mL acetone and 1 g NaClO_2_ was added, and it was heated further for 1 h. This process was repeated for 6–8 h until the lignin was completely removed. The mixture was left for 24 h, and then it was filtered through a tarred and fritted disk glass thimble. The residue was washed with acetone and left in a vacuum oven to dry at 105 °C for 24 h. The solid residue left on the filter gave the weight of holocellulose.

#### 2.2.6. Ash Content (T211 om-16)

For determination, a cleaned, empty crucible was ignited in a muffle furnace at 525 ± 25 °C for 30–60 min, cooled in a desiccator, then weighed. Then 2 g of test specimen was transferred to the crucible and ignited in the muffle furnace at 100 °C, and the temperature was raised to 525 ± 25 °C. After the sample was charred and combusted completely, but not burned, as indicated by the absence of black particles, it was cooled in a desiccator and weighed. The ash content was calculated with the following formula: ash % = A × 100/B, where A is the weight of ash (g) and B is the weight of the test specimen, moisture-free (g).

### 2.3. Pulping Process

Before pulping, WB samples were conditioned in the lab of the factory in Aswan City, Egypt, at a temperature of 23 ± 2 °C and RH of 60 ± 5%. The moisture content of the shavings was measured using an electric oven at 105 °C for 8 h and recorded to be 9.6% and 9.1% in *E. camaldulensis* and *M. sinclairii*, respectively.

Based on oven-dried weight of wood shavings, 200 gm of wood samples from *E. camaldulensis* and *M. sinclairii* were swelled for one day, filtrated, impregnated in sodium hydroxide 5% solution for 4 h at 100 °C, and washed with hot water at 70 °C.

Kraft pulping was conducted in a stainless steel vessel with 3-L capacity under rotation in an oil bath. The conditions used for pulping of WB samples from *M. sinclairii* and *E. camaldulensis* were as follows: active alkalinity 18% and 24%, temperature 165 °C to 175 °C, and reaction time 60 min to 90 min, respectively, and liquid to wood ratio (liquor ratio) of 10:1. The solid residue was removed and washed with hot and cold water until the pH was neutral, and the resulting pulp was screened in a Valley Flat Screen with 0.25 mm slots. Collection of pulp and conventional bleaching stages were carried out with sodium hydroxide and sodium hypochlorite, and excess DW was used for washing between stages (Table 1).

Pulps were evaluated for unscreened and screened yield and kappa number of unbleached pulp (T 236 om-13), as per standard test procedures. Moisture content was determined as per TAPPI test method T 210 cm-13. Freeness of pulp (Schopper-Riegler, SR^0^, Karl Schröder/Kg, D-6940, Weinheim, Germany) was measured according to ISO 5267.

The kappa number (T 236 om-13) is the volume (in milliliters) of 0.1 N potassium permanganate solution consumed by one gram of moisture-free pulp under the conditions specified in this method. The results were corrected to 50% consumption of the permanganate added, and the pulp specimen (1 g) that consumed approximately 50% of the potassium permanganate solution was weighed. At the same time, a second pulp specimen was weighed out to determine the moisture content in accordance with T 550. The pulp sample was disintegrated in 500 mL of DW, then transferred to a 2000 mL reaction beaker, and the apparatus was rinsed out with DW (275 mL) to reach a total volume of 795 mL. The beaker was placed in a bath with constant temperature at 25 ± 0.2 °C during the entire reaction, with stirring. Then 100 ± 0.1 mL of potassium permanganate solution (0.1 N) and 100 mL of sulfuric acid solution (4.0 N) were piped into a 250 mL beaker at 25 °C and immediately added to the disintegrated samples, and simultaneously a stopwatch was started. The beaker was rinsed out using 5 mL of DW. After 10 min, the reaction was stopped with the addition of 20 mL of potassium iodide solution.

The free iodine was titrated with sodium thiosulfate solution and a few drops of starch indicator were added toward the end of the reaction. For blank determination, the same method was carried out but without pulp. The kappa number was calculated with the following formula: K = (p × f)/w; p = [(b − a)N]/0.1, where K is the kappa number, f is a factor (1.002) for correction to 50% permanganate consumption, w is the weight of moisture-free pulp in the specimen (g), p is the amount of 0.1 N permanganate actually consumed by the test specimen (mL), b is the amount of thiosulfate consumed in the blank determination (mL), a is the amount of thiosulfate consumed by the test specimen (mL), and N is normality of the thiosulfate (0.1 N).

The Schopper-Riegler machine was used to determine the freeness of pulp and designed to be given a measure of the rate at which a dilute suspension of pulp (2 g of pulp in 1 L of water) may be drained. The freeness or drainage rate has been shown to be related to the surface conditions and swelling of the fibers.

### 2.4. Extraction of Oily Extracts and GC/MS Analysis 

*Melia azedarach* L. fruits, *Magnolia grandiflora* L. leaves, and *Sinapis alba* L. seeds were used for extraction. About 50 g from each plant material was soaked in a conical flask containing 100 mL of *n*-hexane for 3 days, then filtered through Whatman no. 1 filter paper. The solvent was evaporated using a rotary evaporator and the n-hexane oily extracts (HeOEs) were concentrated. GC/MS analysis was used to analyze the oils with a Focus GC-DSQ Mass Spectrometer (Thermo Scientific, Austin, TX, USA) with a TG-5MS direct capillary column (30 m × 0.25 mm × 0.25 µm film thickness) as reported in the previous work [18]. The Xcalibur 3.0 data system of the GC/MS with threshold values was used to confirm that all MS were attached to the library by measuring the standard index (SI) and reverse standard index (RSI), where a value ≥ 650 was acceptable to confirm the compounds [11,49,50,51].

### 2.5. Addition of Diluted Oily Extracts

A stock solution of 20% of each HeOE was prepared by dissolving 20 mL from each HeOE in 80 mL dimethyl sulfoxide (DMSO) (10%) to a final volume of 100 mL. 0.4, 1.3, and 2.26 mL from each HeOE (20%) concentration were added to 8.64 g of each pulp to reach the concentrations of 1%, 3%, and 5% [Oil/Pulp (OD)], respectively, and mixed well for 1 h before forming of handsheets. The pulp with 8.64 g OD weight from each WB sample of *E. camaldulensis* and *M. sinclairii* was divided to produce 6 sheets. Each 1.44 g sheet was weighed to make a standard sheet of 70 g/m^2^.

### 2.6. Paper Sheet Formation 

Paper sheets with a basis weight (grammage) of 70 g/m^2^ were made using a TAPPI Handsheet Former (code: P41521, Paper Testing Instruments GmbH (PTI Laborausrüstung), Laakirchen, Austria) as per TAPPI test method procedure T 205 sp18. Prior to testing, the handsheets were conditioned for 48 h at 23 °C and 50% RH (ISO 187).

Pulps of 8.64 g were diluted with water to 3000 mL and disintegrated at 3000 rpm, then 500 mL of the disintegrated diluted pulp was taken to make standard sheets (70 g/m^2^) at 22 °C. The paper sheets were made using a sheet machine, where the containers were filled with water and 500 mL of stock was placed. Using rotary oscillation of the stirrer allowed the water to go down in the cylinder in 3 ± 1 s. After that and immediately after the water drained from the sheet, the container was opened and the sheet was picked up with blotting paper, dried, and weighed to calculate the stock concentration to make standard sheets for physical testing (1.44 g). A clean mirror-polished plate was placed on the wet test sheet and covered with another dry blotter ready to receive the next couch blotter and sheet, then the sheets were placed in the press template to make first pressing (5 min at 50 psi). The polished plates were removed and placed in another side, then pressure (50 psi) was applied for 21.30 min as the second pressing. The sheets were removed from the press template and dried at 23 °C and 50% RH using drying rings (T_402_). 

### 2.7. Testing of Mechanical Strength and Optical Properties

Table 2 presents the measured mechanical and optical properties of the paper sheets produced from pulp of WB sample of *E. camaldulensis* and *M. sinclairii*. All experiments were performed in triplicate and the average values are reported [52].

#### 2.7.1. Tensile Index

Tensile index was measured as tensile strength (N/m) divided by grammage (g/m^2^), where 10 test specimens with 15 mm wide were cut from the paper sheet and the jaws of the tensile tester were set 100 mm apart. Tensile index was measured as follows: tensile index (m N/g) = force with Newton/[0.15 × basis weight (70 g/m^2^)].

#### 2.7.2. Burst Index

A circular rubber diaphragm 30.5 mm in diameter was cut from the sheets and the bursting strength was measured by making 10 bursts, one at a time, on the segments of 5 test sheets, where the glazed side of each sheet was clamped toward the diaphragm. The burst index was measured as follows: burst index = (burst, kpa)/basis weight (70 g/m^2^).

#### 2.7.3. Tear Index

Sections from 5 sheets (plies) were cut for a test specimen 53 mm long by 63.0 ± 0.15 mm wide, with tearing all the plies from a single sheet together. The tear index was calculated as follows: tear index (mN·m^2^/g) = (force, mN)/basis weight (70 g/m^2^).

#### 2.7.4. Fold Number 

Folding number tests were used to estimate the paper’s ability to withstand repeated bending, folding, and creasing. A strip of paper 15 mm wide was tested under standard tension of 9.81 N. All test methods were according to the standard TAPPI test methods.

#### 2.7.5. Antifungal Evaluation In Vitro

The produced paper sheets from treated and untreated pulp of *E. camaldulensis* and *M. sinclairii* with the 3 oils were autoclaved before being placed on the medium. Furthermore, for sterilization, fungal inoculation was done inside a laminar flow with the presence of a UV lamp [7]. Discs of paper sheets 9 mm in diameter were inoculated with a fungal disc 5 mm in diameter from *Aspergillus niger* Ani245 and *A. terreus* Ate456 for 14 days at 25 ± 1 °C using PDA culture as growing medium [5,6]. Inhibition zone (mm) and fungal growth on paper discs were recorded [5,6,53].

#### 2.7.6. SEM Examination of Produced Paper Sheets

Scanning electron microscope (SEM) examination was used to show the fungal growth on paper discs taken from those treated and untreated with oils and inoculated with each of the two molds using the JFC-1100E ion sputtering device (model JSM-5300, JEOL, Tokyo, Japan) at 8 kV [5,6].

### 2.8. Statistical Analysis

Data for tensile index, burst index, tear index, double fold number, and brightness were statistically analyzed with three factors (pulp type, oils, and their concentrations) using the SAS system software [54]. Comparisons among means were recorded using Tukey’s studentized range (honest significant difference, HSD) test at alpha 0.05.

## 3. Results and Discussion

### 3.1. Chemical and Physical Analysis of Wood Materials before Pulping and Unbleached Pulp

Soluble contents in cold/hot water, benzene and alcohol extractives, lignin, ash, pentosane, and holocellulose of *E. camaldulensis* and *M. sinclairii* WBs are shown in Table 3. Previously, the chemical composition of *M. sinclairii* stem-wood was 8.0%, 44.0%, 23.2%, 27.9%, and 7.79% total extractives, cellulose, hemicellulose, lignin, and ash, respectively [46], while the present study determined values of 6.32%, 61%, 16%, 23%, and 1.1% for benzene/alcohol extractives, holocellulose, pentosane, lignin, and ash, respectively. The unbleached pulp properties of *E. camaldulensis* and *M. sinclairii* WBs in terms of pulp yield, initial Schopper-Riegler (SR^0^), and kappa number are presented in Table 4.

Previously, pulping of *E. camaldulensis* wood with the kraft method yielded 57.64% [55], while it ranged from 59–62% for *E. grandis* [56]. Lignin, *α*-cellulose, and pentosan contents of pulp from stem wood of *E. camaldulensis* were 12.15%, 70.37%, and 10.96%, respectively [55]. Compared to other *Eucalyptus* species, contents of holocellulose and Klason lignin were 80.5% and 20% in *E. globulus* [57], and 75.2% and 24.8% in *E. grandis*, respectively [58].

### 3.2. Paper Sheet Testing 

**Figure 1:**
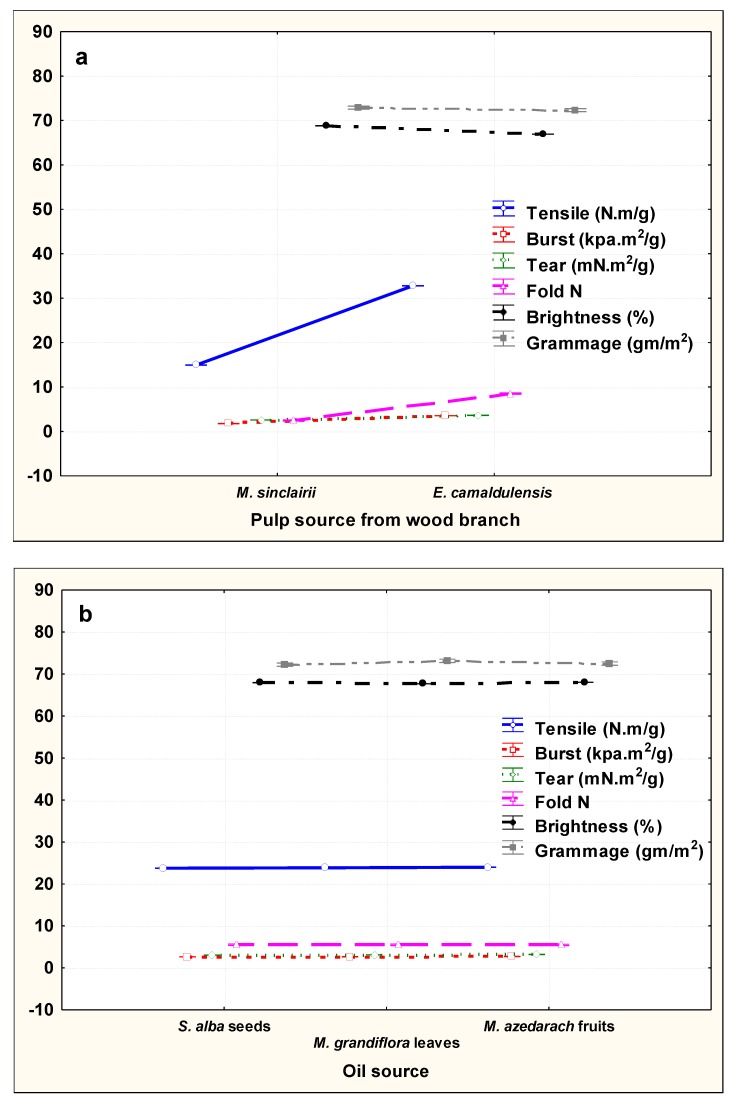

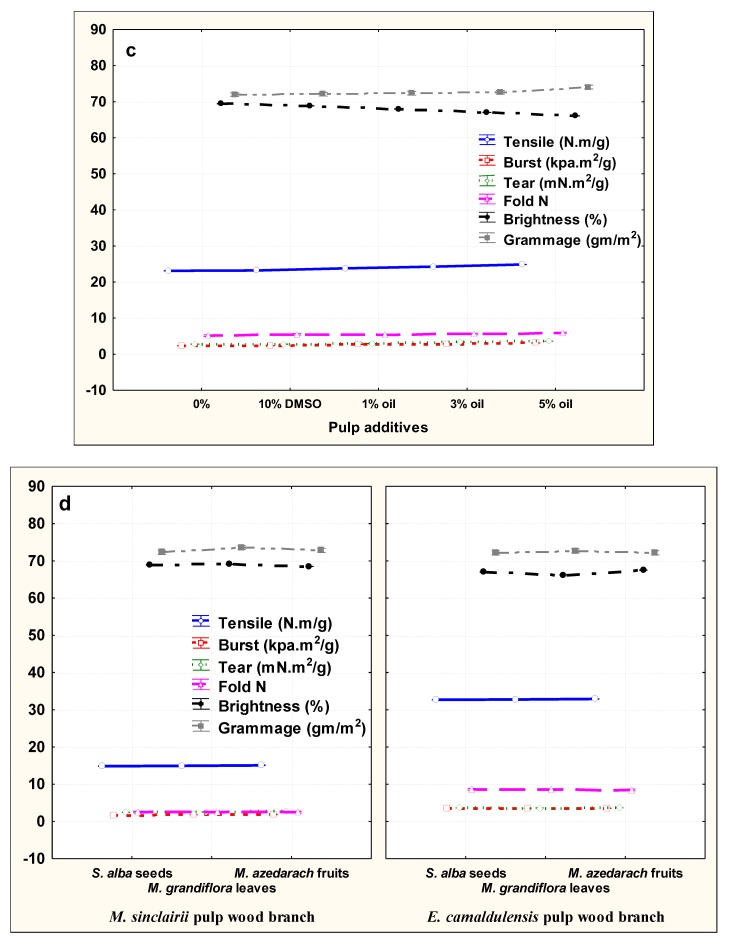

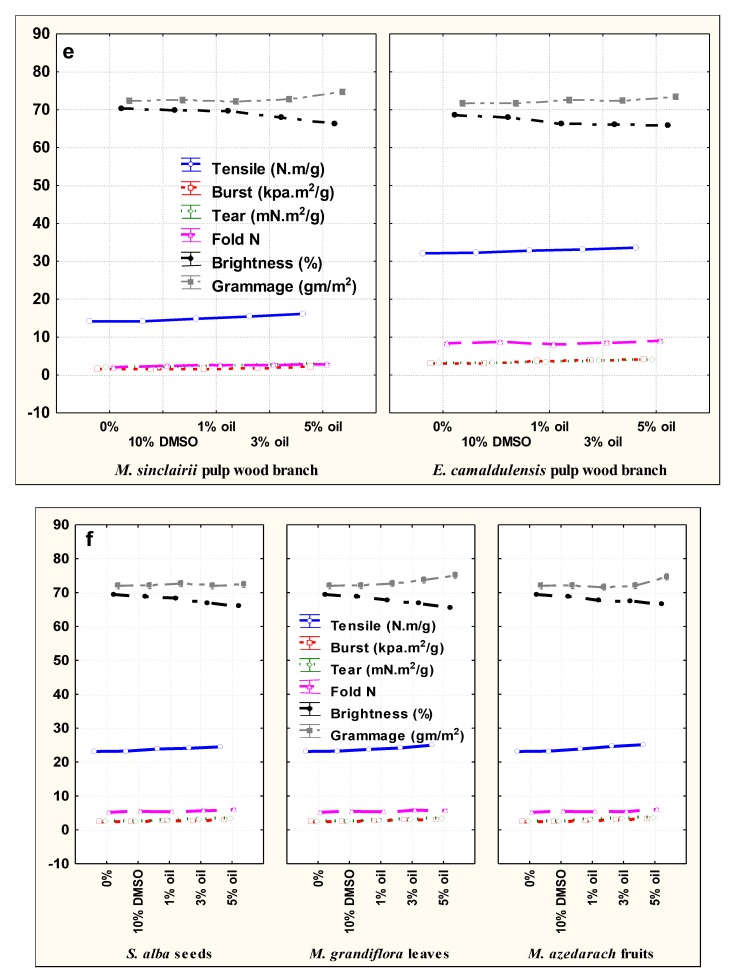
Effects of pulp source, HeOE source, and HeOE concentration on the mechanical and optical properties of the produced paper sheets. (**a**): Pulp source; (**b**): HeOE source; (**c**): HeOE concentrations; (**d**): interaction between pulp source and HeOE source; (**e**): interaction between pulp source and HeOE concentrations; (**f**): interaction between HeOE source and HeOE concentrations.

Statistically, there were overall significant effects from the main factors. Paper sheets produced from *E. camaldulensis* pulp showed the highest mechanical and optical properties compared to those from *M. sinclairii* pulp, while brightness and weight were highest for paper sheets produced from *M. sinclairii* pulp (Figure 1a). The changes in mechanical and optical properties were found to be very small as affected by the addition of n-hexane oily extracts (HeOEs), but HeOE of *S. alba* showed significant effects, followed by *M. grandiflora* and *M. azedarach* HeOEs (Figure 1b). Figure 1c shows that increasing the HeOE concentration (%) led to an increase in the mechanical properties of the produced paper sheets, while the brightness percentage was decreased.

For the interactions between two factors, the mechanical properties of paper sheets of *E. camaldulensis* pulp increased with HeOEs compared to *M. sinclairii* pulp (Figure 1d), as well as with HeOE concentrations (Figure 1e).

For the interactions between HeOEs source and their concentrations, with increased HeOE concentration, the mechanical properties increased, while the brightness percentage decreased (Figure 1f).

Table 5 presents the values of mechanical and optical properties of paper sheets as affected by the three factors (pulp source, HeOE source, and HeOE concentration). Significantly, the tensile index values were higher for paper sheets made from WB pulp of *E. camaldulensis* than those of *M. sinclairii*. The highest values were observed for *E. camaldulensis* WB pulp treated with HeOE of *S. alba* at 5% (33.90 N·m/g) followed by 3% (33.40 N·m/g) and *M. grandiflora* at 5% (33.76 N·m/g) and *M. azedarach* at 5% (33.14 N·m/g) compared to control (without additives, 32.10 N·m/g).

Also, among the HeOE treatments for *M. sinclairii* WB pulp, HeOEs of *S. alba* at 5% and *M. grandiflora* at 5% showed the highest tensile index values at 16.36 N·m/g and 16.20 N·m/g, respectively, compared to control treatment (without additives, 14.14 N·m/g).

For the tear index, *E. camaldulensis* WB pulp treated with 5% HeOEs of *S. alba* (4.11 mN·m^2^/g), *M. azedarach* (4.07 mN·m^2^/g), and *M. grandiflora* (4.06 mN·m^2^/g) showed higher values compared with control (3.32 mN·m^2^/g). Among the *M. sinclairii* WB pulp treatments, the highest values were observed with HeOE of *S. alba* at 5% (3.40 mN·m^2^/g) and 3% (3.10 mN·m^2^/g), compared to control (2.23 mN·m^2^/g).

The highest burst index values were observed for *E. camaldulensis* WB pulp treated with HeOE of *S. alba* at 5% (4.11 kPa·m^2^/g) and 3% (3.92 kPa·m^2^/g) and *M. azedarach* at 5% (3.97 kPa·m^2^/g) compared to control (3.08 kPa·m^2^/g). On the other hand, among the effects of HeOE pulp addition for *M. sinclairii* WB, the highest values were reported with 5% HeOE of *S. alba* (2.56 kPa·m^2^/g) and *M. grandiflora* (2.20 kPa·m^2^/g) compared with control (1.56 kPa·m^2^/g).

Paper sheets made from the pulp of *E. camaldulensis* wood branch showed significantly higher double fold number (DFN) values than those made from pulp of *M. sinclairii* WB. The highest values were observed with *E. camaldulensis* WB pulp treated with 5% HeOEs of *S. alba*, *M. azedarach*, and *M. grandiflora*, where the DFN reached 9 compared to control (8.33). For WB pulp from *M. sinclairii*, the highest values were reported with treatment of 5% HeOEs from *S. alba*, *M. azedarach*, and *M. grandiflora* where the DFN reached 3 compared to control (2). 

Paper sheets produced from *M. sinclairii* WB pulp without additives showed the highest brightness percentage (70.31%), followed by pulp treated with 1% HeOEs of *M. azedarach* (70.23%) and *M. grandiflora* (69.92%). 

Paper sheets produced from WB pulp of *M. sinclairii* treated with 5% HeOEs of *S. alba* and *M. grandiflora* showed the highest grammage values of 75.96 and 75.30 g/m^2^, respectively, followed by those treated with 3% HeOE of *M. grandiflora* (74.47 g/m^2^).

Previous burst index (kPa·cm^2^/g) and tear index (mN·cm^2^/g) values of paper tested with *E. camaldulensis* were 1.18 and 2.15, respectively [55]. Paper sheets tested from pulp of 27 clones of *Eucalyptus* trees, including *E. camaldulensis*, *E. teriticornis*, and *E. urophylla*, showed a tensile index of 35–54 N/mg, burst index of 1.9–3.3 kPa·m^2^/g, and tear index of 3.5–5.6 N·m^2^/g [59]. Paper sheets from *E. camaldulensis* stem wood produced with kraft pulping showed a brightness value of 79.0% [55].

### 3.3. Evaluation of Antifungal Activity of Paper Sheets 

**Figure 2:**
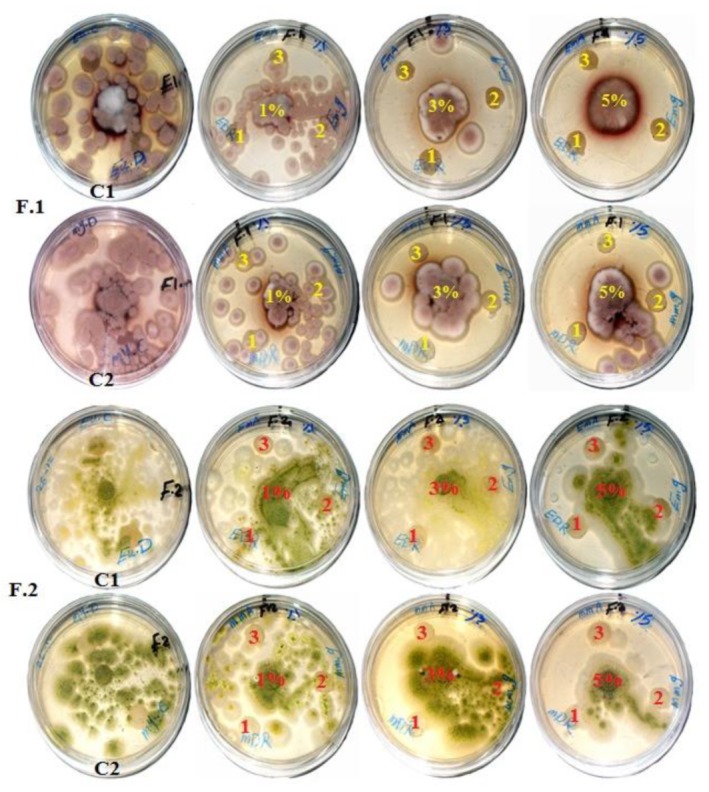
Fungal growth 14 days after inoculation and incubation of pulp paper discs produced with three HeOEs as additives. C1: Control of paper discs from *E. camaldulensis* treated with 10% DMSO; C2: control of paper discs from *M. sinclairii* treated with 10% DMSO; F1: *A. terreus* (first and second rows); F2: *A. flavus* (third and fourth rows); 1: HeOE of *S. alba* seeds; 2: HeOE of *M. grandiflora* leaves; 3: HeOE of *M. azedarach* fruits.

The antifungal activity of paper discs made from pulp of *E. camaldulensis* and *M. sinclairii* WBs with different concentrations of the three HeOEs as additives is presented in Table 6. The inhibition zones (IZs) of fungal linear growth observed around the paper discs are shown in Figure 2. Compared with control, IZs ranged from 3 to 7 mm and from 0.5 to 5 mm against the growth of *Aspergillus terreus* on pulp paper discs of *E. camaldulensis* and *M. sinclairii* WBs, respectively, with HeOEs of *M. azedarach*, *S. alba*, and *M. grandiflora* at 3% and 5%.

For growth of *A. flavus*, paper discs produced from pulp of *M. sinclairii* WB showed IZs of 1 and 4 mm for pulp treated with 3% and 5% HeOE *M. azedarach* fruits, respectively. Paper discs from *E. camaldulensis* WB pulp treated with all the studied HeOEs did not show any IZ against *A. flavus* and there were various degrees of growth of the fungus on the paper discs.

### 3.4. SEM Examination of Inoculated Paper Sheets

#### 3.4.1. Fungal Infestation of Inoculated Paper Sheets

**Figure 3:**
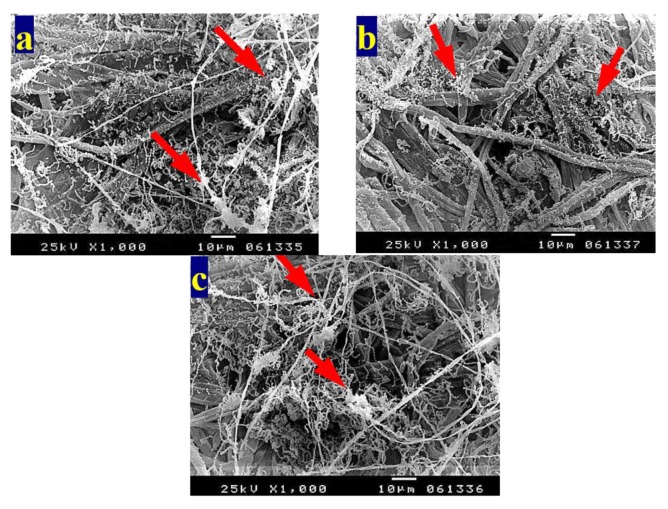
SEM images of *E. camaldulensis* WB pulp paper manufactured without HeOE additives and inoculated with *A. terreus*: (**a**,**b**) without additives, where arrows refer to dense growth of fungal mycelia and fungal spores on sample fibers; and (**c**) with 10% DMSO where arrows refer also to huge agglomeration of fungal mycelia on the surface of the sample fibers.

**Figure 4:**
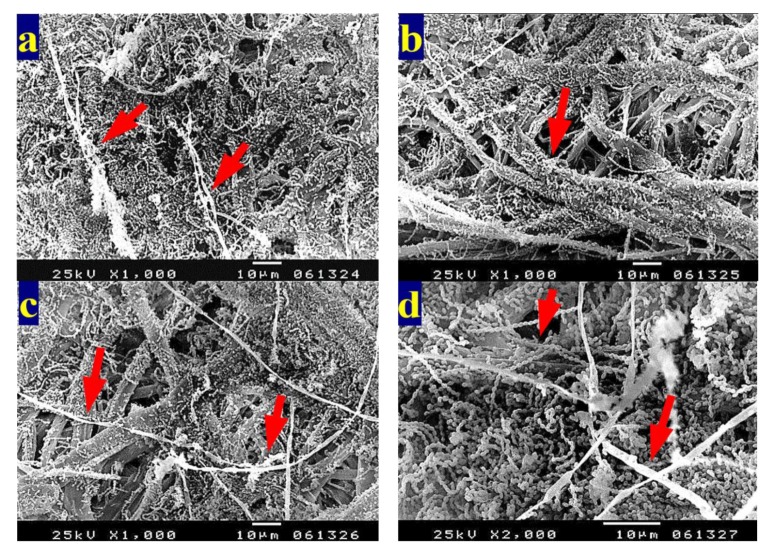
SEM images of *E. camaldulensis* WB pulp paper manufactured with HeOE additives and inoculated with *A. terreus*: (**a**) with *M. azedarach* oil at 1%, where arrows indicate a dense fungal spores and mycelia covering the surface of the fibers; (**b**) with *S. alba* at 1%, where arrow refers to huge fungal spores encapsulates the fibers, and (**c**,**d**) with *M. grandiflora* oil at 1%, where arrows refer to dense growth of fungal mycelia and spores that covers the entire surface of the sample almost completely.

Figure 3 shows the patterns of decay caused by *A. terreus* on *E. camaldulensis* WB paper sheets without pulp additives (Figure 3a,b) or with 10% DMSO (Figure 3c). SEM images show a huge growth of *A. terreus* as well as penetration of fungal hyphae within the fiber bundle and fungal growth covering the fibers. These results are in accordance with Taha et al. [6], who found huge growth of *A. terreus* on pulp paper produced from linen fibers without additives. HeOEs at 1% did not show any decease in fungal growth, where intensive growth of fungal mycelia of *A. terreus* was observed on pulp treated with HeOE from *M. azedarach* (Figure 4a), *S. alba* (Figure 4b), and *M. grandiflora* (Figure 4c,d).

**Figure 5:**
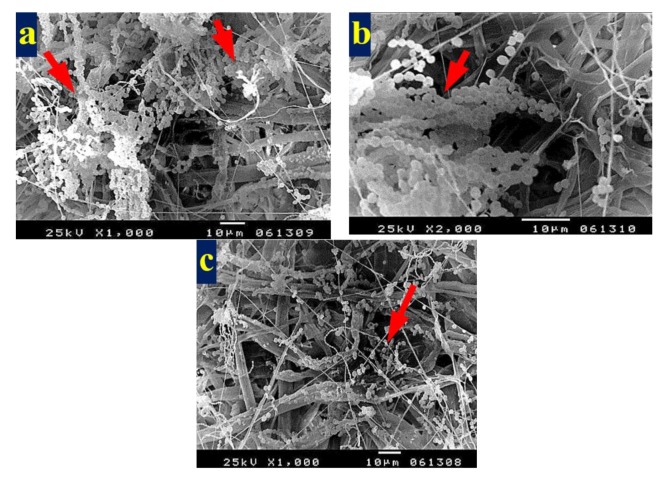
SEM images of *E. camaldulensis* WB pulp paper manufactured without HeOE additives and inoculated with *A. flavus*: (**a**,**b**) without additives and arrows indicate the magnitude of the fungal spores scattered on the surface of the sample fibers, and (**c**) treated with 10% DMSO, where arrow refers to dense growth of fungus mycelia on and between sample fibers.

**Figure 6:**
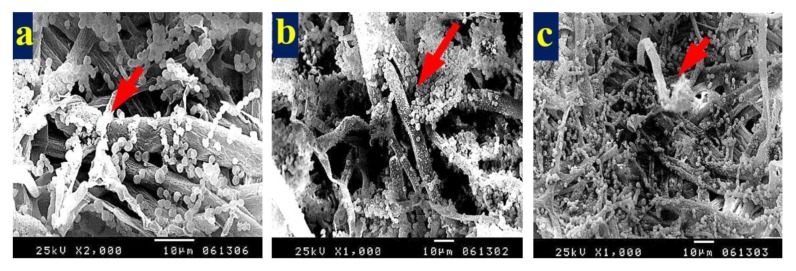

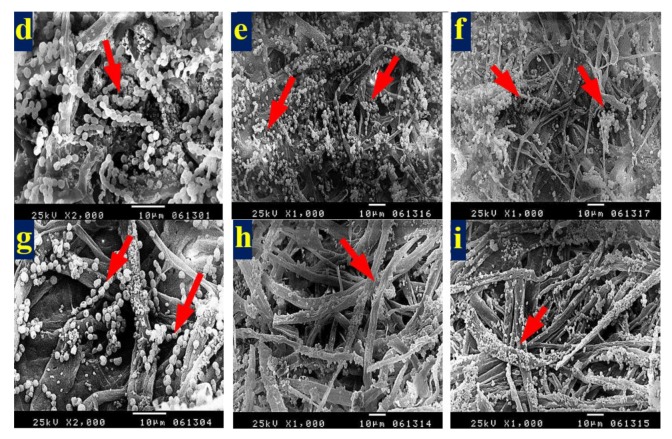
SEM images of *E. camaldulensis* WB pulp paper manufactured with the addition of different HeOEs and inoculated with *A. flavus*: (**a**) with *M. azedarach* HeOE at 1%; (**b**) with *S. alba* HeOE at 1%; (**c**) with *M. grandiflora* HeOE at 1%; (**d**) with *M. azedarach* HeOE at 3%; (**e**) with *S. alba* HeOE at 3%; (**f**) with *M. grandiflora* HeOE at 3%; (**g**) with *M. azedarach* HeOE at 5%; (**h**) with *S. alba* HeOE at 5%; (**i**) with *M. grandiflora* HeOE at 5%. Arrows in (**a**,**d**,**g**) indicate the growth and density of the fungal spores on the sample fibers, arrows in (**b**,**e**,**h**) indicate the growth and interaction of the fungal spores with the fibers, and arrows in (**c**,**f**,**i**) refer to a slightly moderate growth of fungal mycelia and spores on the sample fibers.

Similarly, the dense growth of *A. flavus* for *E. camaldulensis* paper sheets manufactured without additives (Figure 5a,b) and with 10% DMSO (Figure 5c). Additionally, dense growth was shown on pulp treated with HeOEs from *M. azedarach* at 1% (Figure 6a), *S. alba* at 1% (Figure 6b), and *M. grandiflora* at 1% (Figure 6c). There was a slight decrease in *A. flavus* growth on pulp treated with HeOEs from *M. azedarach* at 3% (Figure 6d), *S. alba* at 3% (Figure 6e), and *M. grandiflora* at 3% (Figure 6f). A reasonable decrease of *A. flavus* growth was observed on pulp treated with HeOEs from *M. azedarach* at 5% (Figure 6g), *S. alba* at 5% (Figure 6h), and *M. grandiflora* at 5% (Figure 6i).

**Figure 7:**
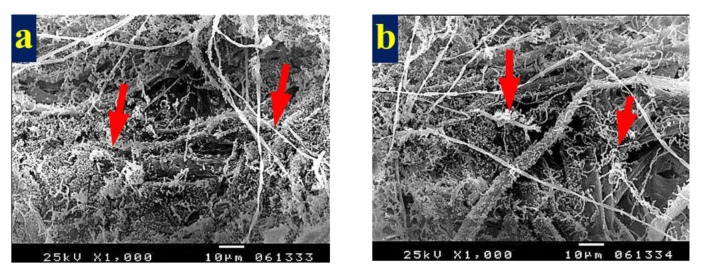

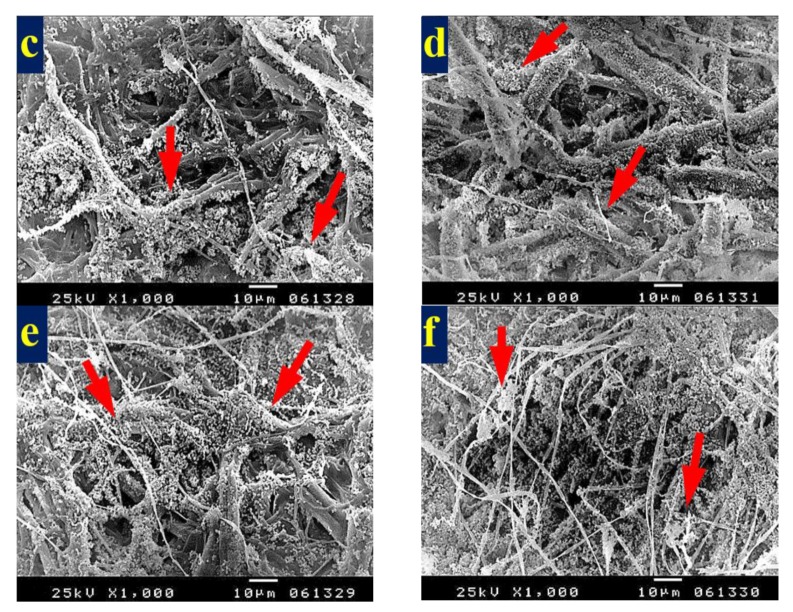
SEM images of *M. sinclairii* WB pulp paper manufactured with/without HeOE additives and inoculated with *A. terreus*: (**a**) without HeOE additives; (**b**) with 10% DMSO; arrows in (**a**,**b**) indicate the prevalence and penetration of fungal mycelia and spores in the sample fibers; (**c**,**d**) with *M. azedarach* HeOE at 1%; (**e**) with *S. alba* HeOE at 1%; (**f**) with *M. grandiflora* HeOE at 1%, where arrows in (**c**–**f**) indicate the fungal spores that cover most of the sample fibers and a lower prevalence of the fungal mycelia.

Paper sheets produced from *M. sinclairii* WB pulp without additives showed dense growth of *A. terreus* mycelium (Figure 7a) with 10% DMSO (Figure 7b), with HeOEs from *M. azedarach* at 1% (Figure 7c,d), with *S. alba* at 1% (Figure 7e), and with *M. grandiflora* at 1% (Figure 7f).

**Figure 8:**
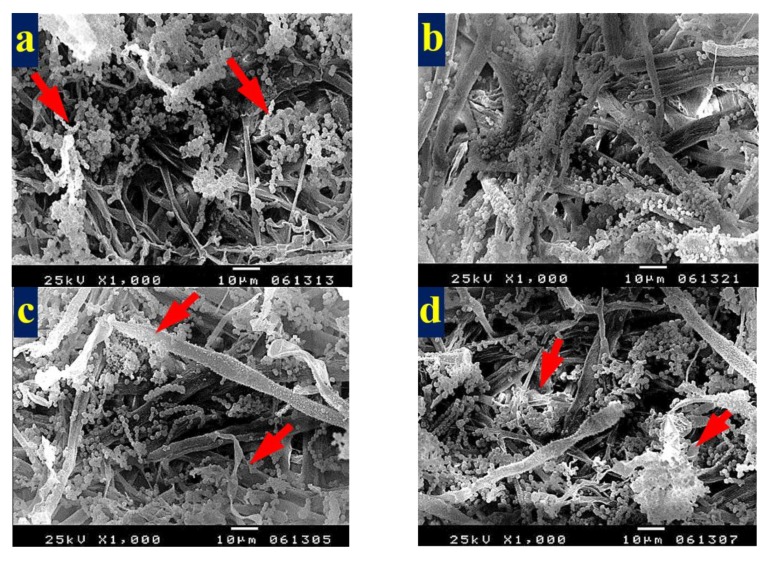

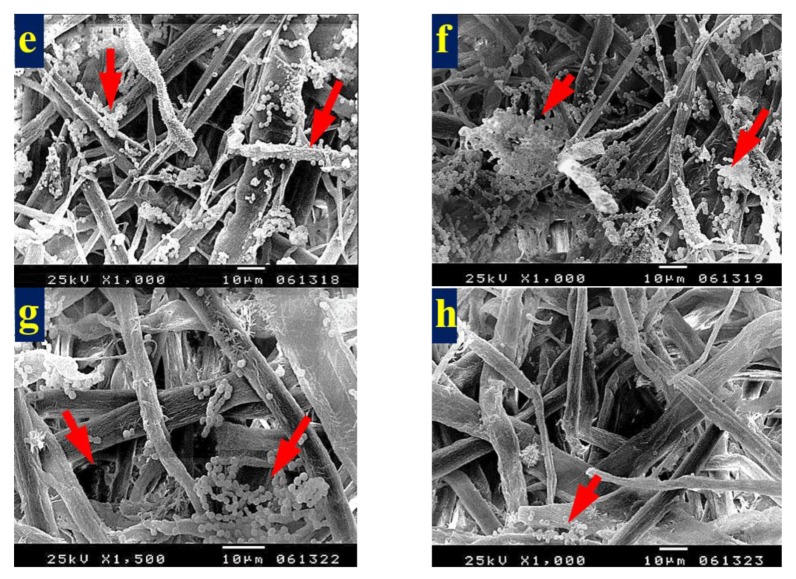
SEM images of *M. sinclairii* WB pulp paper produced with/without HeOE additives and inoculated with *A. flavus*: (**a**) without HeOE additives; (**b**) with 10% DMSO; arrows in (**a**,**b**) indicate fungal mycelia and spores prevalence and penetration especially on sample fibers; (**c**) with *M. azedarach* HeOE at 1%; (**d**) with *M. grandiflora* HeOE at 1%; arrows in (**c**,**d**) indicate the intensity of the fungal mycelia growth and spores diffusion; (**e**) with *M. azedarach* HeOE at 3%; arrows in (**e**,**f**) indicate spores diffusion in and between fibers; (**f**) with *M. grandiflora* HeOE at 3%; (**g**) with *S. alba* HeOE at 5%; (**h**) with *M. grandiflora* HeOE at 5%, where arrows in (**g**,**h**) indicate a marked decrease in the density of the fungal mycelia growth and decrease in the fungal spores based on type and concentration of added HeOEs.

*A. flavus* showed dense growth on pulp paper of *M. sinclairii* WB without HeOE additives (Figure 8a), with 10% DMSO (Figure 8b), with *M. azedarach* HeOE at 1% (Figure 8c), with *M. grandiflora* HeOE at 1% (Figure 8d), with *M. azedarach* HeOE at 3% (Figure 8e), and with *M. grandiflora* HeOE at 3% (Figure 8f). On the other hand, a decrease in the growth of fungal mycelia and the appearance of cell walls of cellulosic fibers were found on pulp treated with HeOE from *S. alba* at 5% (Figure 8g) and *M. grandiflora* at 5% (Figure 8h).

#### 3.4.2. Inhibition of Fungal Growth of Inoculated Paper Sheets

**Figure 9:**
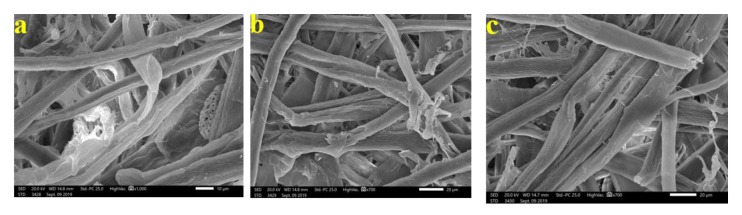

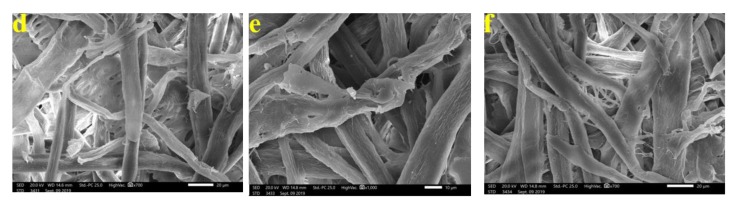
SEM images shows inhibition of fungal growth on *M. sinclairii* WB pulp paper produced with the addition of HeOEs and inoculated with *A. terreus*: (**a**) with *M. azedarach* HeOE at 3%; (**b**) with *M. azedarach* HeOE at 5%; (**c**) with *S. alba* HeOE at 3%; (**d**) with *S. alba* HeOE at 5%; (**e**) with *M. grandiflora* HeOE at 3%; (**f**) with *M. grandiflora* HeOE at 5%.

**Figure 10:**
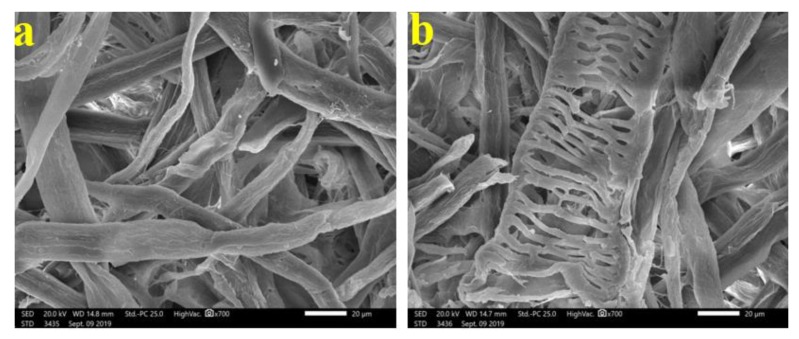
SEM images show inhibition of fungal growth on *M. sinclairii* WB pulp paper produced with the addition of HeOEs and inoculated with *A. flavus*: (**a**) with *M. azedarach* HeOE at 5%; (**b**) with *M. grandiflora* HeOE at 5%.

**Figure 11:**
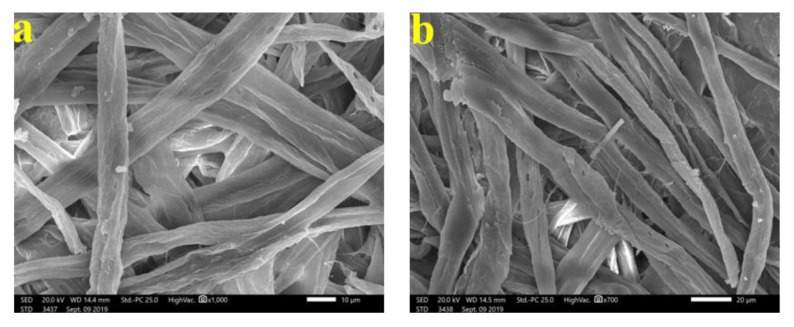

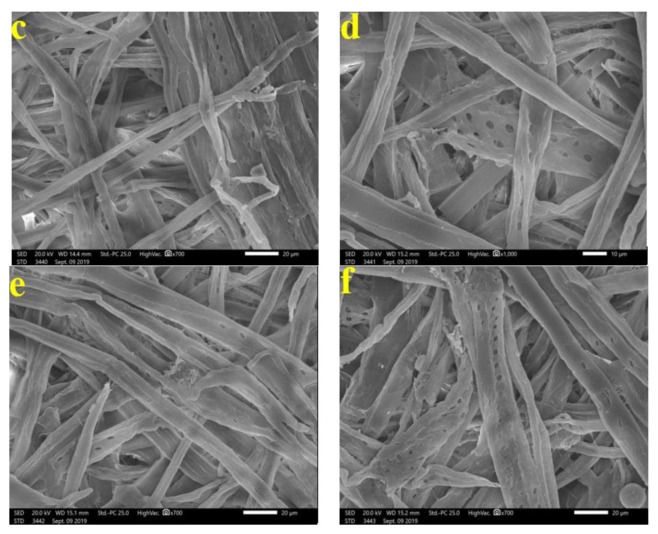
SEM images show inhibition of fungal growth on *E. camaldulensis* WB pulp paper manufactured with the addition of HeOEs and inoculated with *A. terreus*: (**a**) with *M. azedarach* HeOE at 3%; (**b**) with *M. azedarach* HeOE at 5%; (**c**) with *S. alba* HeOE at 3%; (**d**) with *S. alba* HeOE at 5%; (**e**) with *M. grandiflora* HeOE at 3%; (**f**) with *M. grandiflora* HeOE at 5%.

**Figure 12:**
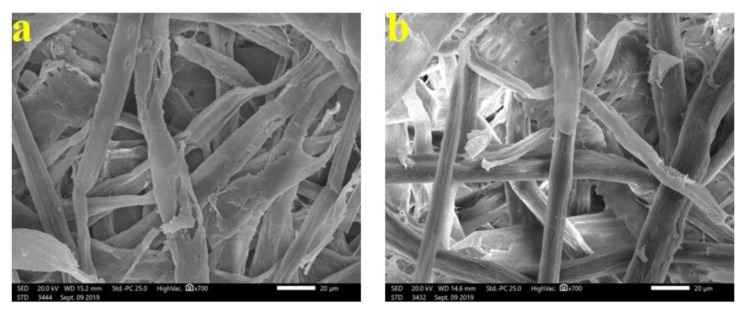
SEM images show inhibition of fungal growth on *E. camaldulensis* WB pulp paper manufactured with HeOE additives and inoculated with *A. flavus*: (**a**) with *M. azedarach* HeOE at 5%; (**b**) with *S. alba* HeOE at 5%.

To show the fungal growth inhibition of inoculated paper sheets with the studied HeOEs, SEM examination was done. No growth of *A. terreus* (Figure 9a–f) and *A. flavus* (Figure 10a,b) was shown for *M. sinclairii* WB pulp paper produced with the addition of the studied HeOEs at 3% or 5%. The same was found with paper sheets produced from *E. camaldulensis* WB pulp treated with the HeOEs at 3% and 5% and inoculated with *A. terreus* (Figure 11a–f) and *A. flavus* (Figure 12a,b). Moreover, long fibers with less porosity and very good structure can be seen in Figure 9, Figure 10, Figure 11 and Figure 12.

### 3.5. Chemical Composition of the Oils

According to the results of fungal inhibition of paper sheets produced from pulp treated with the HeOEs, the activity could be related to the presence of the chemical constituents of the HeOEs. Therefore, GC/MS was done to identify the chemical components. The main chemical compounds of *S. alba* seed HeOE were oleic acid (12.35%), eicosadienoic acid (9.66%), linolenic acid (8.94%), campesterol (7.72%), palmitelaidic acid (7.42%), and palmitic acid (7.07%) (Table S1). Another study reported that the major fatty acids in mustard were erucic, linolenic, and linoleic acids, with values of 29.81%, 23.92%, and 23.57%, respectively [26]. The main triacylglycerol components in the lipid composition of five accessions of *S. alba* were erucic, oleic, palmitic, gadoleic, and linoleic acids with amounts of 28.0–53.2%, 13.7–25.1%, 3.9–5.2%, 9.4–14.2%, and 4.9–17.4%, respectively, while the sterol fractions were *β*-sitosterol (51.9–55.9%) followed by campesterol (19.1–30.5%) and brassicasterol (11.9–22.5%) [60]. Other studies reported that the oil contains a high level of tocopherols (α-, β-, γ-, and δ-tocopherols are also established in mustard) [61,62]. Oleic acid (7.8%), linoleic acid (3.2%), and palmitic acid (3.1%) were identified in *n*-hexane oil extract from *S. alba* [63] that at 10 µL showed activity against the growth of *A. niger*, *A. flavus*, *Fusarium monoliforme*, *F. graminearum*, and *Penicillium viridicatum*.

Palmitic acid (7.28%), oleic acid (7.22%), undecane (5.37%), palmitoleic acid (4.66%), (1-propyloctyl) benzene (4.63%), (1-methyldecyl)-benzene (4.21%), (1-ethylnonyl) benzene (3.88%), (1-propylnonyl) benzene (3.86%), stearic acid (3.46%), (1-pentylhexyl) benzene (3.36%), (1-ethyldecyl) benzene (3.3%), and linoleic acid (3%) were the main compounds present in the HeOE of *M. grandiflora* leaves (Table S2). The majority of fatty acids in leaves of the Magnoliaceae family are palmitic, palmitoleic, steric, oleic, linoleic, and eleostearic [64]. The essential oil was found to have *γ*-elemene (15.7%), 2,6-dimethyl-6-bicyclo[3.1.1]hept-2-ene (11.6%), caryophyllene (9.0%), and spathulenol (6.5%) as the main compounds [65].

The main constituents identified in *M. azedarach* fruit HeOE were oleic acid (10.73%), hexanoic acid (9.72%), hexadecanol (6.61%), (1-methylnonadecyl)-benzene (5.66%), linoleic (5.24%), palmitic acid (5.18%), (1-butyloctyl)-benzene (5.16%), dodecane (5.02%), (1-pentyloctyl)-benzene (5.02%), and linolenic acid (4.08%) (Table S3).

Oil from ripe fruits of *M. azedarach* showed the presence of oleic acid, hexadecanol, myristic acid methyl ester, palmitic acid, linoleic acid methyl ester, oleic acid methyl ester, and stearic acid methyl ester in amounts of 15.16%, 3.07%, 1.00%, 6.77%, 34.72%, 32.45%, and 6.83%, respectively [29]. Stearic, palmitic, palmitoleic, oleic, and linoleic acids, which known to be potential antifungal agents, were the main fatty acid compounds in the ethanolic extract of fruits [66,67]. Linolenic, linoleic, and oleic acids exhibited antifungal activity [68]. Methyl esters of fatty acids from palmitic (18.8%), methyl linolenic (16.1%), and linoleic (9.8%) acids were identified in the *n*-hexane oil extract of fruits [69].

Overall, the oil additives used for pulp from *E. camaldulensis* and *M. sinclairii* wood branches enhanced the mechanical properties of the produced papersheets, and significantly, the antifungal activities of the produced papersheets were much improved with increasing the oils’ concentrations.

## 4. Conclusions

In the present work, natural oily extracts from *S. alba* seeds, *M. azedarach* fruits, and *M. grandiflora* leaves were used as pulp additives to improve the mechanical, optical, and antifungal properties of paper sheets produced from *E. camaldulensis* and *M. sinclairii* wood branches. The antifungal potential of oils at 3% and 5%, with no fungal growth of *A. terreus* and *A. flavus*, was demonstrated. This is probably due to the main chemical compounds in these oils. In addition, the mechanical properties of paper sheets produced from *E. camaldulensis* pulp were significantly better than those from *M. sinclairii* pulp. Slight but significant enhancements in mechanical properties of paper sheets produced from the pulp of *E. camaldulensis* and *M. sinclairii* wood branches were observed as the concentration of oils was increased.

## Figures and Tables

**Table 1:** Conventional bleaching conditions of wood branch (WB) pulp from *E. camaldulensis* and *M. sinclairii*.

Bleaching Conditions	*E. camaldulensis*	*M. sinclairii*
1. Sodium hydroxide stage		
Sodium hydroxide %, for 2 h at 75 °C	2	2
Initial brightness	24	28
2. Hypochlorite stage		
Hypochlorite %, for 3 h at 35 °C	4	4
Final brightness	68.56	70.31

**Table 2:** Standard test methods used for mechanical strength and optical properties of bleached initial pulp obtained from samples of *M. sinclairii* and *E. camaldulensis* wood species.

Paper Sheet Properties	Standard Method	Model Machine Used
Tear index, mN·m^2^/g	T 414 om-12	FRANK-PTI GMBH, Elmendorf tear tester, model 53984, serial no. 40551
Tensile index, N m/g	T 404 wd-03	Adamel Lhomargy, model 596420, DY-30, Saint-Baldoph, France
Burst index, kPa m^2^/g	T 403 om-15	Tecnolab Company, model BS 20 E, serial no. 160.08, Laakirchen, Austria
Double fold number	T 511 om-13	Folding endurance, KÖGEL LEIPZIG, DFP 6/60 9401
Brightness, %	T525 om-17	Color Touch Model ISO, model CTH-ISO, serial no. CTH A 2054, Technidyne Corporation, New Albany, IN, USA

**Table 3:** Chemical and moisture content analyses of *E. camaldulensis* and *M. sinclairii* shavings from wood branches before pulping.

Analysis	Value in Wood-Branch Shavings	
	*E. camaldulensis*	*M. sinclairii*
Solubility in cold water	4.45%	6.95%
Solubility in hot water	10.2%	11.3%
Benzene and alcohol extractives	8.92%	6.32%
Acid-insoluble lignin (Klason lignin)	27%	23%
Wood shaving ash	0.9%	1.1%
Pentosans	14%	16%
Holocellulose	56%	61%
Moisture content	9.6%	9.1%

**Table 4:** Unbleached pulp characteristics after cooking process.

Pulp Properties	Wood-Branch Pulp	
	*E. camaldulensis*	*M. sinclairii*
Pulp yield (%)	48	37
Initial Schopper-Riegler, SR^0^	27	19
Kappa number	24	18

**Table 5:** Effects of different HeOEs with their concentrations on mechanical and optical properties of the produced paper sheets from pulps of *E. camaldulensis* and *M. sinclairii* branch wood.

Wood Pulp	HeOE Additives	Conc.	Mechanical Properties				Optical Properties	Grammage g/m^2^
			Tensile Index	Tear Index	Burst Index	Double Fold	Brightness	
			N·m/g	mN·m^2^/g	kPa·m^2^/g	No.	%	
*E. camaldulensis*	Without additive	0%	32.10 ± 0.17 *	3.32 ± 0.005	3.08 ± 0.04	8.33 ± 0.57	68.56 ± 0.057	71.65 ± 0.49
	DMSO	10%	32.26 ± 0.15	3.34 ± 0.01	3.12 ± 0.01	8.66 ± 0.57	67.87 ± 0.005	72.31 ± 0.28
	*S. alba*	1%	32.86 ± 0.02	3.72 ± 0.005	3.54 ± 0.01	8.00	66.35 ± 0.03	71.82 ± 0.28
		3%	33.40 ± 0.17	3.86 ± 0.01	3.92 ± 0.06	8.33 ± 0.57	67.13 ± 0.05	73.31 ± 1.59
		5%	33.90 ± 0.05	4.11 ± 0.11	4.11 ± 0.01	9.00	67.63 ± 0.11	72.98 ± 0.28
	*M. azedarach*	1%	32.87 ± 0.005	3.44 ± 0.005	3.76 ± 0.005	8.33 ± 0.57	66.5 4 ± 0.005	72.48 ± 0.28
		3%	32.98 ± 0.005	3.87 ± 0.005	3.87 ± 0.01	8.66 ± 0.57	66.17 ± 0.112	71.98 ± 0.76
		5%	33.14 ± 0.049	4.07 ± 0.06	3.97 ± 0.01	9.00	65.76 ± 0.057	72.15 ± 0.86
	*M. grandiflora*	1%	32.69 ± 0.16	3.44 ± 0.02	3.66 ± 0.005	8.00	65.66 ± 0.057	72.98 ± 1.52
		3%	32.98 ± 0.01	3.81 ± 0.06	3.87 ± 0.02	8.66 ± 0.57	64.89 ± 0.011	74.97 ± 0.28
		5%	33.76 ± 0.005	4.06 ± 0.063	3.98 ± 0.01	9.00	64.19 ± 0.06	71.65 ± 0.49
*M. sinclairii*	Without additive	0%	14.14 ± 0.16	2.23 ± 0.02	1.56 ± 0.005	2.00	70.31 ± 0.025	72.31 ± 0.28
	DMSO	10%	14.16 ± 0.057	2.26 ± 0.015	1.58 ± 0.02	2.33 ± 0.57	69.81 ± 0.106	72.48 ± 0.28
	*S. alba*	1%	14.83 ± 0.11	2.55 ± 0.005	1.75 ± 0.005	2.66 ± 0.57	68.98 ± 0.01	70.99 ± 3.76
		3%	15.80 ± 0.10	3.10 ± 0.10	1.96 ± 0.017	2.33 ± 0.57	67.65 ± 0.005	72.32 ± 0.28
		5%	16.36 ± 0.057	3.40 ± 0.17	2.56 ± 0.32	3.00	65.73 ± 0.05	75.96 ± 0.76
	*M. azedarach*	1%	14.83 ± 0.11	2.27 ± 0.011	1.61 ± 0.005	2.33 ± 0.57	70.23 ± 0.005	72.32 ± 0.28
		3%	15.16 ± 0.057	2.56 ± 0.011	1.67 ± 0.015	2.66 ± 0.57	67.46 ± 0.21	71.49 ± 0.28
		5%	15.83 ± 0.057	2.94 ± 0.011	1.96 ± 0.011	3.00	66.60 ± 0.10	72.81 ± 0.28
	*M. grandiflora*	1%	14.80 ± 0.10	2.43 ± 0.01	1.65 ± 0.011	2.66 ± 0.57	69.92 ± 0.05	73.31 ± 2.91
		3%	15.33 ± 0.11	2.87 ± 0.01	1.87 ± 0.01	3.00	68.73 ± 0.057	74.47 ± 1.03
		5%	16.20 ± 0.10	2.98 ± 0.01	2.20 ± 0.17	2.33 ± 0.57	66.60 ± 0.10	75.30 ± 1.43
Minimum significant difference **			0.367	0.154	0.224	1.476	0.235	3.487

Notes: *: Values are presented as mean ± SD; S.A., *S. alba*; M.A., *M. azedarach*; M.G., *M. grandiflora*; HeOE, n-hexane oily extract; DMSO, dimethyl sulfoxide. **: The minimum significant difference according to Tukey’s studentized range (honest significant difference, HSD) test.

**Table 6:** Screening of antifungal activity of produced paper sheets against growth of *A. terreus* and *A. flavus.*

WB Pulp	HeOE	Conc.	*A. terreus*		*A. flavus*	
			Inhibition Zone (mm)	Growth on Disc (mm)	Inhibition Zone (mm)	Growth on Disc (mm)
			14th day		14th day	
*E. camaldulensis*	No additives	0	0	5–7	0	10
	DMSO	10%	0	6–7	0	10
	*M. azedarach*	1%	0	1–3	0	1–2
		3%	3–7	0	0	0–2
		5%	5–7	0	1	0
	*S. alba*	1%	0	3–8	0	2–3
		3%	2–5	0	0	0.5–2
		5%	3–7	0	0.5	0–1
	*M. grandiflora*	1%	0	8–9	0	8–9
		3%	2–6	0	0	7–8
		5%	3–7	0	0	1–4
*M. sinclairii*	No additives	0	0	9–10	0	3–8
	DMSO	10%	0	9–10	0	4–8
	*M. azedarach*	1%	0	9–10	0	3–4
		3%	1–3	0	1	0
		5%	3–5	0	4	0
	*S. alba*	1%	0–2	0–3	0	0–1
		3%	1–4	0	0	0–1
		5%	2–3	0	0	0–1
	*M. grandiflora*	1%	0	0.5–2	0	5–6
		3%	0–2	0–0.5	0	3–9
		5%	0.5–2	0	0–2	0–1

Notes: HeOE: *n*-hexane oily extract; Inhibition zones were recorded without adding the disc diameter [43]; S.A., *S. alba;* M.A., *M. azedarach;* M.G., *M. grandiflora.*

## Supplementary Materials

The following are available online at https://www.mdpi.com/1996-1944/13/6/1292/s1, Table S1: Chemical composition of HeOE from *S. alba* seeds, Table S2: Chemical composition of HeOE from *M. grandiflora* leaves, Table S3: Chemical composition of HeOE from *M. azedarach* fruits.
