# Supplementary material for: Impact of Three Natural Oily Extracts as Pulp Additives on the Mechanical, Optical, and Antifungal Properties of Paper Sheets Made from Eucalyptus camaldulensis and Meryta sinclairii Wood Branches
Source: Materials (Basel). 2020 Mar 12;13(6):1292. doi: 10.3390/ma13061292 (PMC7142441; doi:10.3390/ma13061292)
Supplement: Supplementary file 1 [file materials-13-01292-s001.pdf]

materials

Article

Impact of Three Natural Oily Extracts as Pulp Additives on the Mechanical, Optical, and Antifungal Properties of Paper Sheets Made from *Eucalyptus camaldulensis* and *Meryta sinclairii* Wood Branches

Mohamed Z.M. Salem [1,*], Wael A.A. Abo Elgat [2], Ayman S. Taha [3], Yahia G.D. Fares [4], and Hayssam M. Ali [5,*]

[1] Forestry and Wood Technology Department, Faculty of Agriculture (EL-Shatby), Alexandria University, Alexandria 21545, Egypt
[2] Restoration Department, High Institute of Tourism, Hotel Management and Restoration, Abukir, Alexandria 21526, Egypt; watsat20@yahoo.com
[3] Conservation Department, Faculty of Archaeology, Aswan University, Aswan 81528, Egypt; aymansalahtaha82@yahoo.com
[4] Laboratory and Research, Misr Edfu Pulp Writing and Printing Paper Co. (MEPPCO), Aswan 81656, Egypt; yahyagml@yahoo.com
[5] Botany and Microbiology Department, College of Science, King Saud University, P.O. Box 2455, Riyadh 11451, Saudi Arabia
* Correspondence: zidan_forest@yahoo.com (M.Z.M.S.); hayhassan@ksu.edu.sa (H.M.A.)

Received: 25 February 2020; Accepted: 9 March 2020; Published: date

Table S1. Chemical composition of HeOE from *S. alba* seeds.

Compound	Percentage in Oil (%)	SI	RSI
Geranyl vinyl ether	1.75	739	763
cis-9,10-Epoxy-octadecanoic acid	4.56	765	781
Palmitelaidic acid	7.42	780	792
Z-(13,14-epoxy)tetradec-11-en-1-ol acetate	1.42	776	796
Oleic acid	12.35	827	830
7-Methyl-Z-tetradecen-1-ol acetate	0.76	788	812
Linolenic acid	8.94	684	857
Linoleic acid	4.06	700	785
2'-Hexyl-1,1'-bicyclopropane-2-octanoic acid methyl ester	3.61	791	839
2-Methylene-5α-cholestan-3β-ol	3.76	790	837
β-Sitosterol	5.56	686	811
cis-9,10-Epoxystearic acid	5.37	779	808
Campesterol	7.72	666	672
Palmitic acid	7.07	710	780
Eicosadienoic acid	9.66	706	781
α-Tocopherol	8.30	700	734
Arachidic acid	1.74	870	900
Erucic acid	4.90	666	892

SI, standard index; RSI, reverse standard index.

Table S2. Chemical composition of HeOE from *M. grandiflora* leaves.

Compound	Percentage in Oil (%)	SI	RSI
Undecane	5.37	928	951
(*Z*)-9-Octadecenoic acid	1.69	759	759
2-Methyl-undecane	1.64	805	835
Tetradecyl-oxirane	2.22	774	836
(*Z*)-10-Pentadecen-1-ol	0.82	749	792
Oleic acid	7.22	926	932
2-Methyldecalin	1.25	823	872
17-Octadecynoic acid	0.55	763	763
5-Nonadecen-1-ol	0.87	782	798
1-Tetradecanol	1.57	783	786
2-Methyl-dodecane	1.22	774	810
2-[12-(Oxiran-2-yl)dodecyl]oxirane	0.73	747	751
β-Elemene	1.07	859	880
(1-Butylhexyl)benzene	2.74	893	909
(1-Propylheptyl)benzene	2.15	896	914
(1-Pentylhexyl)benzene	3.36	876	888
Palmitic acid	7.28	901	910
(1-Propyloctyl)benzene	4.63	918	923
(1-Ethylnonyl)benzene	3.88	905	913
(1-Pentylheptyl)benzene	5.22	905	915
(1-Methyldecyl)-benzene	4.21	904	915
(1-Propylnonyl)benzene	3.86	913	923
(1-Ethyldecyl)benzene	3.30	876	896
Palmitoleic acid	4.66	842	886
Linoleic acid	3.00	868	887
Stearic acid	3.46	901	924
(1-Propyldecyl)benzene	2.20	851	865
Spathulenol	2.66	804	876
(1-Methyldodecyl)benzene	2.04	881	882
Nonacosane	1.03	801	807

SI, standard index; RSI, reverse standard index.

Table S3. Chemical composition of HeOE from *M. azedarach* fruits.

Name	Percentage in Oil (%)	SI	RSI
Hexanal	3.99	678	903
Hexanoic acid	9.72	674	899
1,1'-(1,2-Dimethyl)-cyclohexane	0.78	668	889
E-2-Tetradecen-1-ol	0.47	680	900
Tetradecane	3.43	666	843
Dodecane	5.02	668	669
2,6-Dimethyl-undecane	1.16	861	870
2,4-Decadienal	2.31	668	670
(1-Butylhexyl)- benzene	2.86	864	875
Myristic acid methyl ester	2.21	681	684
(1-Ethyloctyl)-benzene	1.67	873	890
Pulegol	0.34	685	702
Stearic acid methyl ester	2.64	670	686
Oleic acid	10.73	882	806
Palmitic	5.18	850	916
(1-Ethylnonyl)-benzene	3.57	728	750
Hexadecanol	6.61	710	771
Linoleic acid	5.24	707	709
(1-Butyloctyl)-benzene	5.16	783	873
Linolenic	4.08	783	815
(1-Ethyldecyl)-benzene	3.02	893	911
(1-Methylnonadecyl)-benzene	5.66	758	790
(1-Pentyloctyl)-benzene	5.02	789	804
(1-Butylnonyl)-benzene	3.22	787	797
(1-Propyldecyl)-benzene	2.39	783	868
Tetracosanoic acid	1.83	773	878
trans-9-Octadecenoic acid	1.70	771	791

SI, standard index; RSI, reverse standard index.

© 2020 by the authors. Submitted for possible open access publication under the terms and conditions of the Creative Commons Attribution (CC BY) license (http://creativecommons.org/licenses/by/4.0/).
